# A novel prediction approach of three-dimensional thermal fatigue cracks in thermal compression bonding electrodes based on digital twin

**DOI:** 10.1371/journal.pone.0339003

**Published:** 2025-12-16

**Authors:** Qianyu Ren, Kai Yang, Zuoen Deng, Jiadui Chen, Haisong Huang, Jingwei Yang

**Affiliations:** 1 Key Laboratory of Advanced Manufacturing Technology of the Ministry of Education, Guizhou University, Guiyang, China; 2 School of Mechatronic Engineering and Automation, Foshan University, Foshan, China; Galgotias University, INDIA

## Abstract

Thermal compression bonding (TCB) electrodes that initiate thermal fatigue cracks compromise reliability and takt time in electronic manufacturing, and accurate prediction of three-dimensional (3D) electrode cracks is a prerequisite for crack mitigation. This study developed a digital twin (DT) framework that combined physics-based simulation and artificial intelligence (AI). The framework used the extended finite element method (XFEM) to build a high-fidelity electrode DT and reproduced fatigue behavior under coupled electrical, thermal, and mechanical loading through adaptive updating. To alleviate the scarcity of crack data, a conditional variational autoencoder (CVAE) with a position attention (PA) mechanism was constructed, with an error of 0.7% to 1.3% relative to experimental results. Using the augmented data, the PA-RePointNet model was developed to predict 3D crack morphology. Results showed that PA-RePointNet surpassed PointNet++ and PointCNN in prediction accuracy and stability and achieved a mean absolute error (MAE) of 2.8, a root mean square error (RMSE) of 5.1, and a coefficient of determination (R²) of 0.9378, while the maximum relative error between the reconstructed 3D cracks and experimental measurements was 1.87%. This framework provides a high-precision solution for electrode crack prediction and opens a new pathway for intelligent maintenance of TCB electrodes in microelectronic manufacturing.

## Introduction

As microelectronic packaging advances toward higher integration density, higher power density, and higher reliability, the long-term stability of interconnect structures has become a critical bottleneck limiting product quality and reliability. Thermal compression bonding (TCB) is an advanced soldering technology in which Joule heating generated by an electric current in opposing electrodes locally raises the temperature at the bonding interface while the electrodes simultaneously apply a compressive load to form a metallurgical joint [1]. Owing to its high throughput, small heat-affected zone (HAZ), rapid thermal transients, and controllable deformation, TCB is widely used where extremely tight alignment and connection tolerances are required, including 2.5D/3D electronic packaging [2], flexible printed circuit (FPC) bonding, and coil bonding [3,4]. However, as device integration and power densities continue to rise, the TCB process places increasingly stringent demands on the thermal stability and structural reliability of the electrodes. In service, electrodes experience cyclic, coupled electro-thermo-mechanical fields [5], which render them susceptible to the initiation of thermal-fatigue cracks. Crack growth degrades effective thermal conductivity and induces spatially non-uniform bonding temperatures, which in turn cause defects such as cold solder joints, inadequate wetting, and joint offset. In severe cases, once cracks reach a critical length, electrode fracture or electrical arcing can occur, jeopardizing equipment safety and risking production interruptions. Real-time, accurate monitoring of TCB electrode cracking is therefore essential for consistent bonding quality and overall process reliability.

Although direct studies on crack detection in TCB electrodes are scarce, extensive research on crack initiation and evolution in mechanical engineering provides a strong theoretical and practical foundation. Traditional nondestructive testing (NDT) methods remain core approaches for crack identification, each offering domain-specific advantages. Ultrasonic testing (UT) employs acoustic wave propagation and precise localization to differentiate and quantify internal defects. For example, Xia et al. [6] used through-thickness ultrasonic penetration to differentiate and measure weld-root cracks in orthotropic steel bridge decks, and Yan et al. [7] established a probability of detection curve for railway hollow axle cracks based on UT, which offers a quantitative basis for crack assessment of critical components. Radiographic testing (RT) can visually present crack morphology and size through imaging. Schulze [8] demonstrated its utility for detecting internal structural defects in civil-engineering applications. Eddy-current testing (ET) leverages electromagnetic induction and excels at detecting surface and near-surface cracks in conductive metals. For instance, Xia et al. [9] developed an ET method based on magnetic-induction intensity for detecting cracks in steel bridge decks, and Yuan et al. [10] used direct-current electromagnetic NDT to determine the orientation of rolling-contact-fatigue (RCF) cracks in moving ferromagnetic materials. While these methods can periodically detect macroscopic cracks, they generally require complex procedures and manual operation, limiting online, real-time monitoring and early warning of fracture risk. Moreover, their sensitivity to incipient damage is low, making it difficult to observe the full crack-evolution process from initiation to propagation. Achieving real-time sensing and dynamic prediction across the entire crack-growth process remains a key bottleneck for traditional detection technologies.

The convergence of digital twin (DT) technology and artificial intelligence (AI) offers new opportunities for real-time monitoring and state prediction in manufacturing systems. By constructing high-fidelity virtual models that are bidirectionally mapped to their physical counterparts, and by using AI algorithms to assimilate streaming sensor data and perform intelligent inference, DT enables state awareness, performance optimization, and predictive control of complex manufacturing processes [11–13]. In recent years, DT-AI frameworks have also been widely applied to fatigue-crack prediction and structural health monitoring. Kim et al. [14] proposed a physics-informed DT method based on Lamb-wave monitoring and Bayesian model updating, achieving dynamic identification and life prediction of fatigue cracks in lap joints by inverting sensor responses. Tian et al. [15] constructed a DT for turbine-blade crack growth from two-dimensional temperature and stress fields, integrating automated detection, model updating, and life prediction with a prediction error of 7.15%. Anvari et al. [16] combined experimental data from carbon-fiber-reinforced-polymer (CFRP) and aluminum adhesively bonded joints under hygrothermal aging with machine learning in a DT framework for online modeling, enabling real-time fatigue-life prediction and quantification of environmental degradation effects. Chen et al. [17] used online strain measurements to build a probabilistic framework that integrates DT with a dynamic Bayesian network (DBN), providing real-time estimates of crack growth and remaining life. Cheok et al. [18] proposed a strain-interface DT with Bayesian updating to correct the growth rate of corner fatigue cracks in real time, substantially improving remaining-life prediction. He et al. [19] developed a DT framework based on Bayesian entropy using corrosion-fatigue image data to predict the life of bridge suspension cables and to quantify uncertainty. Collectively, these studies show that synergistic DT-AI coupling markedly improves the accuracy of crack-behavior prediction. Recent work also indicates that optimization-based approaches for manufacturing and reliability prediction can further enhance model adaptability and robustness [20]. Nevertheless, for TCB electrodes operating under extreme coupled electrical, thermal, and mechanical conditions, high-value monitoring data are scarce; because high-quality, diverse data are foundational for DT models and AI training, advances in data-augmentation techniques are urgently needed.

In this context, generative AI has emerged as an important means to address data scarcity. Generative adversarial network (GAN) [21] and variational autoencoder (VAE) [22] are powerful frameworks for producing high-quality, diverse synthetic samples that remain consistent with governing physical constraints. GAN-based models perform especially well for image synthesis, whereas VAEs offer advantages for signal modeling; both families and their variants have achieved notable success in practice. Shin et al. [23] combined multimodal welding images with a GAN for online crack identification and stable spatiotemporal video reconstruction, markedly improving the real-time reliability of process monitoring. Maeda et al. [24] used a progressive-growing GAN (PG-GAN) to generate realistic road-pothole images, effectively enlarging training sets and improving accuracy and robustness in road-damage detection. Han et al. [25] introduced a multi-stage road crack image generation model based on WGAN-GP, ensuring rapid and consistent generation of high-quality images. Pei et al. [26] proposed a method based on an improved deep convolutional GAN (DCGAN) to generate realistic pavement distress images. Ramatlo et al. [27] employed VAE in ultrasonic guided wave monitoring to generate synthetic data for challenging-to-obtain damage scenarios in welded rails, successfully capturing complex physical signal characteristics, such as multimodal propagation and multiple reflections. Therefore, the development of advanced generative AI methods that deeply integrate domain knowledge, simulate infrequent events, and ensure the physical validity of generated data will constitute a crucial breakthrough for alleviating the shortage of electrode crack data.

In summary, although DT and AI have substantially advanced structural health monitoring and crack prediction, most studies rely on one-dimensional signals or two-dimensional images; research on three-dimensional (3D) cracks remains limited. High-quality 3D crack data for electrodes subjected to high-temperature electro-thermo-mechanical coupling are extremely scarce, severely restricting the training and generalization of conventional DT models. To address this gap, a DT-based prediction framework was developed for TCB electrodes that integrates twin data with generative AI to achieve high-fidelity reconstruction and prediction of 3D cracks. The main contributions are as follows:

1. A high-fidelity virtual-physical mapping of 3D electrode crack-growth behavior was achieved using a digital-twin framework.
2. A conditional variational autoencoder (CVAE) [28] with a position attention (PA) [29] mechanism, termed PA-CVAE, was proposed to generate and augment 3D crack-growth data under extreme conditions.
3. An inverse point-cloud model with PA, termed PA-RePointNet, was developed to capture twin-data features and to predict 3D thermomechanical fatigue cracks in TCB electrodes with high accuracy.

The remainder of this paper is organized as follows. In the methodology section, a multi-physics and multi-scale electrode DT is constructed with a self-updating crack simulator based on the extended finite element method (XFEM). The PA-CVAE for current-signal augmentation is introduced, and the PA-RePointNet architecture for 3D crack prediction is presented. In the results and analysis section, simulation outputs are compared with infrared thermography and thermocouple measurements. Crack initiation and propagation are examined. The augmentation quality of PA-CVAE is evaluated. The predictive accuracy and the 3D surface-reconstruction performance of PA-RePointNet are quantified. The discussion section synthesizes insights, limitations, and implications. The conclusion section summarizes the principal findings and outlines directions for future work.

## Methodology

### Electrode DT model construction method

#### Twin model theoretical framework.

**Fig 1:**
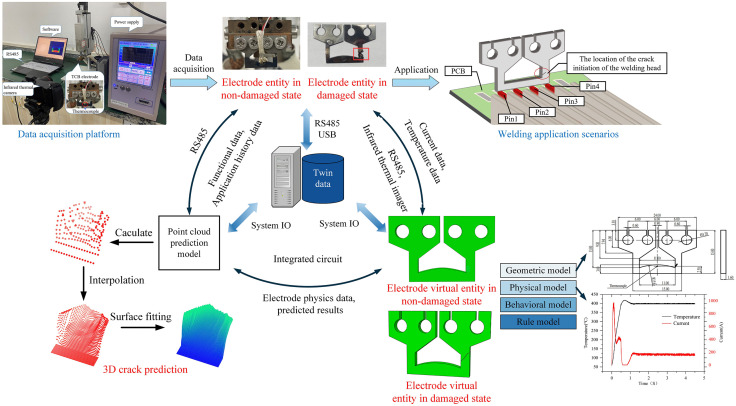
DT framework.

To enable the prediction of cracks under thermal fatigue damage conditions, this study constructed a DT model framework, as illustrated in Fig 1. This framework comprises the electrode’s physical entity, virtual entity, a crack prediction module, twin data, and their connections. The physical entity component was based on the data acquisition platform depicted in Fig 1. This platform integrated a precision inverter TCB power supply, a servo-controlled pressurization system, and a sensing and acquisition system. It was equipped with data output functionality, enabling the post-welding output of critical welding parameters and current profiles at the electrode thermocouple locations, for both non-damaged and damaged states, via an RS485 serial port. Furthermore, an infrared thermal imager (FLIR A615) was employed to capture the electrode’s temperature distribution, providing foundational data for the subsequent development of the virtual model. As depicted in the upper right of Fig 1, the welding application scenario for the physical entity involved the soldering of 0.3 mm² multi-strand copper cables with a 1.8 mm pitch and 4 pins onto a Printed Circuit Board (PCB) featuring tin-plated solder pads.

The virtual entity was constructed using Abaqus finite element software and encompassed four dimensions: geometry, physics, behavior, and rule. The virtual entity performed dynamic simulations using real-time data from the physical entity and continuously self-updated iteratively, ensuring a high degree of synchronization with the actual state of the physical electrode. The 3D crack prediction was primarily based on DT data. From the predicted crack surface point cloud, 3D surface reconstruction was performed, enabling the prediction of electrode crack propagation to guide welding applications of the physical entity. Twin data, comprising physical entity data, virtual entity data, and 3D crack data, was stored on a Dell Precision 3650 Tower workstation. Virtual entity data and 3D crack data were accessed via system I/O calls, while physical entity data was transmitted via an RS485 serial port or USB cable.

#### Virtual model construction method.

The lower right of Fig 1 illustrates the electrodes virtual entity, which comprises geometric, physical, behavioral, and rules models. The geometric model defined the electrode’s physical shape and structural features, providing a foundation for subsequent physical simulation, such as heat conduction and crack propagation. The physical model integrated the electrode’s physical properties, environmental constraints, and key characteristic information within the geometric model, thereby endowing it with realistic attributes and operating conditions. The TCB electrode material was fabricated by wire electrical discharge machining (WEDM) from a pure molybdenum plate (99.95% Mo content), and its physical parameters are detailed in Table 1. Environmental constraints included installation conditions, welding pressure requirements, and the ambient temperature range. Characteristic information included the welding current, applied voltage, and the resultant Joule heating during the welding process.

**Table 1:** Physical properties of the electrode material.

Physical parameter	Value	Physical parameter	Value
Tensile strength	345 MPa	Electrical conductivity	1.87 × 10^7 S/m
Yield strength	240 MPa	Young’s modulus	329 GPa
Melting point	2610 °C	Poisson’s ratio	0.324
Density	10.28 g/cm³	Specific heat capacity	278 J/(kg·K)
Thermal conductivity	142.35 W/(m·K)	–	–

The behavioral model characterized the electrode’s physical behavior during welding by integrating dynamic response data from the physical entity under specific operating conditions and incorporating physical laws. In this study, the electrode behavioral model was constructed using real-time temperature and current data obtained from the data acquisition platform of the physical system. The experiments were conducted under optimal welding parameters, including a welding pressure of 32 N, a welding temperature of 420 °C, a heating-up time of 500 ms, and a holding time of 4000 ms. The core of this model involved simulating the transient changes in the electrode’s internal temperature field due to resistive heating upon energization, thermal stress from coupled temperature gradients and external welding pressure, and consequent deformation and potential damage. Simulation of these dynamic behaviors ensured the virtual model could respond in real-time to changes in the physical electrode.

The rules models defined the criteria and evolutionary logic for the electrode’s transition from normal operation to damage and eventual failure. This model, crucial for crack prediction, integrates physical phenomena with engineering judgment rules to guide the virtual model on when and how to initiate crack propagation simulations. The stress intensity factor (SIF) is a mechanical parameter indicating stress intensity in this region and is crucial for studying crack propagation. Cracks are typically categorized into three types based on the relationship between applied stress and crack displacement: type I cracks, type II cracks, and type III cracks. Among these, type I cracks, characterized by tensile stress perpendicular to the crack plane, causing the crack faces to open, are the most prevalent in engineering scenarios. Furthermore, as the crack propagation characteristics in the TCB electrode predominantly align with this type, only type I cracks were considered in this research. The SIF of type I cracks is commonly denoted as K_I_, and the formula used is as follows:


$$\begin{array}{c} K_{I}=\sigma \sqrt{\pi \alpha } \end{array}$$ (1)

Where σ represents tensile stress and α denotes the crack length.

It can be inferred from Eq (1) [30] that when either σ or α, or both, increase, K_I_ rises correspondingly, indicating an increase in stress components at the crack tip. When K_I_ reaches a certain critical value, stress within a sufficiently large range at the crack tip reaches the fracture strength of the material crack will undergo unstable propagation, resulting in material fracture. This critical K_I_ value is designated as the fracture toughness K_IC_. Based on the relative magnitude relationship between the SIF of the material and the fracture toughness, the K_I_ criterion for unstable crack propagation causing brittle fracture can be established. Since plane strain fracture is the most perilous, the fracture criterion is typically established with K_IC_ as the benchmark, as shown in Eq (2) [30].


$$\begin{array}{c} K_{\text{I}}\geq K_{\text{IC}} \end{array}$$ (2)

When a crack progresses to meet the conditions for fracture failure, brittle fracture occurs. If the specified conditions are not met, fracture failure will not occur, even in the presence of cracks. This situation is called a fail-safe. The simulation process control in this paper is based on this theory.

#### Crack simulation strategy based on a virtual model self-updating.

During the thermal fatigue crack propagation process in the TCB electrode, current, temperature, and stress vary with the evolving crack morphology. To ensure a high degree of consistency between the virtual model’s state and that of the physical entity, this study proposed a Python-based model self-updating method. The core of this method involves the effective integration of XFEM-based crack propagation simulation with iterative updates to the cracked component model. Before this, the initial current and crack parameters were obtained. The initial current was defined as the actual current data measured for the uncracked electrode under optimal welding parameters; this data provided thermal input to the established electrode model. The initial crack information was determined by the evolution trends of thermal strain and maximum principal strain within the TCB electrode during the welding process. Consequently, the electrode’s temperature field was first simulated based on the initial current. The results of this temperature field simulation were then introduced as a thermal load into the stress simulation model, enabling precise localization of the initial crack initiation site. Subsequently, these current and crack parameters were input into the electrode’s XFEM simulation model to perform the 3D crack propagation simulations.

**Fig 2:**
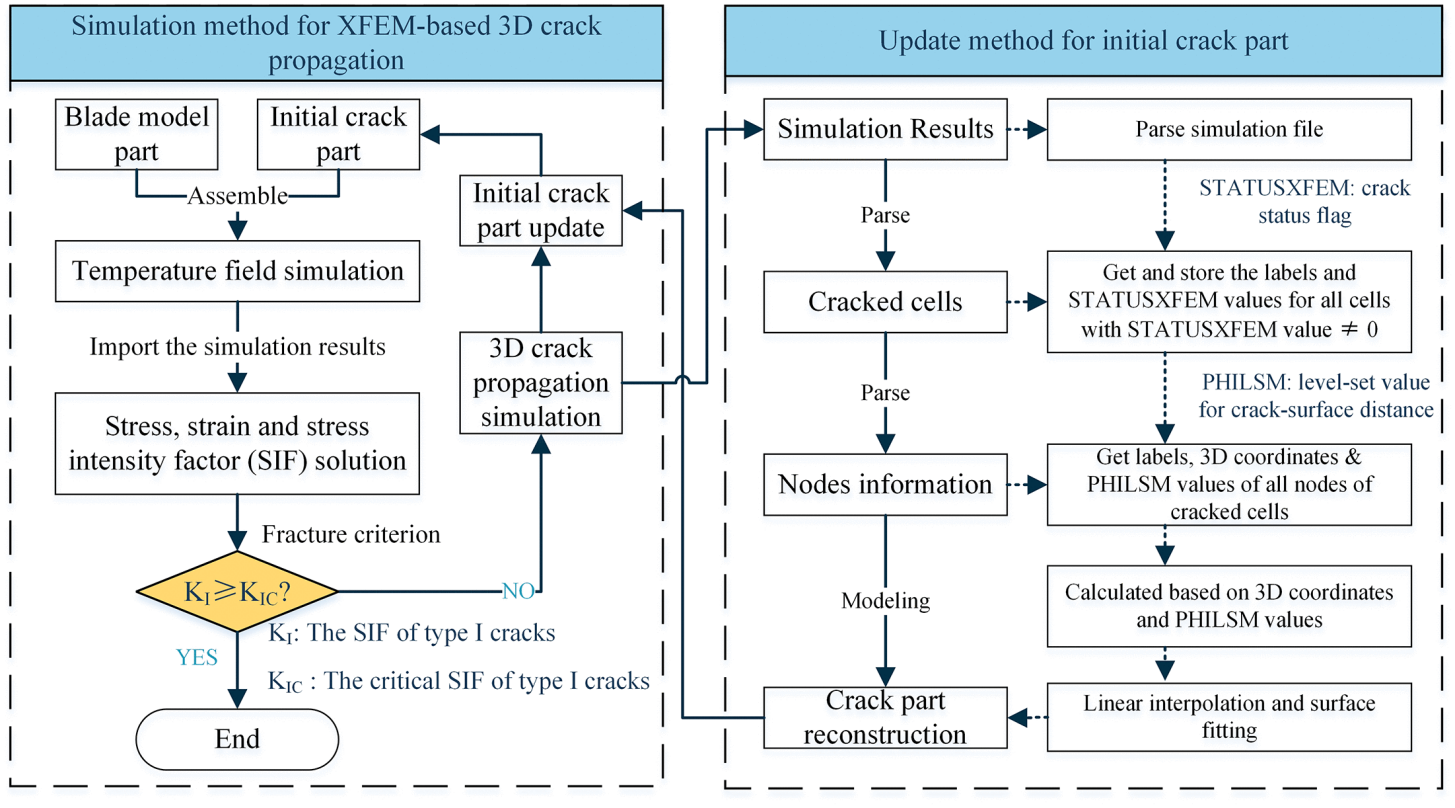
Flowchart of TCB electrode thermal fatigue crack simulation and update method.

The left panel of Fig 2 details the crack simulation procedure, which included the following steps: (1) After determining the initial crack location, an initial component incorporating the predefined crack was established, assembled with the electrode model, and subjected to the defined current load. Subsequently, the temperature field was re-simulated. Based on the resultant temperature field, stress and strain simulations were conducted, along with the calculation of the SIF at the crack tip. (2) If the simulation results at a given stage did not meet the fracture criterion, it indicated that electrode fracture had not yet occurred. In that case, the cracked component was reconstructed based on the observed crack propagation using the proposed component updating method. This reconstructed component was then reassembled with the electrode model to initiate the subsequent simulation cycle. (3) This iterative process was repeated until the fracture criterion was met, at which point electrode fracture was deemed to have occurred, and the simulation was terminated.

As illustrated in the right panel of Fig 2, the detailed workflow for initial crack reconstruction comprised the following four stages: First, simulation output files were parsed to identify elements affected by cracks accurately. The crack propagation status of each component is quantified using the STATUSXFEM variable, which ranges from 0 (intact) to 1 (fully fractured), with intermediate values reflecting varying degrees of crack development. Second, for identified cracked elements, nodal information, including node labels, spatial coordinates, and PHILSM value, was extracted. PHILSM denotes the signed-distance level-set field for the crack surface. When a crack intersects an element edge, the PHILSM values at the two end nodes exhibit opposite signs, and the sum of their absolute values equals unity. Third, based on this principle, crack point cloud data were generated via linear interpolation. By utilizing the PHILSM values and 3D coordinates of nodes along each element edge, the exact intersection points between the crack surface and element edges were calculated. Finally, these point cloud data were interpolated and fitted to form a continuous 3D surface mesh. This reconstructed crack surface was then reassembled with the electrode model, enabling autonomous model updating during the fatigue process. The entire reconstruction pipeline was automated using Python scripts, ensuring both geometric accuracy and efficiency in the simulation workflow.

Notably, during actual welding, variations in crack morphology significantly influence the welding current. Consequently, welding current is utilized as a critical indicator for electrode crack prediction. To establish a fatigue-induced current dataset for predictive modeling, a series of TCB electrodes with identical geometries was fabricated. Each of these electrodes incorporated a prefabricated initial defect of varying length. These initial defects were precisely introduced at predetermined crack initiation sites using wire-cutting technology with a tungsten wire with a 0.2 mm diameter. Subsequently, controlled welding experiments were conducted under optimized process parameters, applying different initial temperatures to electrodes with distinct defect lengths. During each experiment, welding current data were synchronously recorded and later utilized to support the development and training of the crack prediction model.

### Current data augmentation method for electrodes in the damaged state

However, although the proposed model’s self-updating method effectively accommodates temperature and stress variations due to changes in crack morphology, real-time updating of welding current data remains a challenge. Given the advantages of the VAE model in signal data processing, this study introduces a VAE to optimize the existing self-updating framework, enabling dynamic current data updates. The VAE model constructs a generative framework that maps latent variables Z to target data X. Specifically, it assumes that Z follows a predefined probability distribution and learns a transformation from the latent space to the data space corresponding to the target distribution. The process of inferring latent vectors Z from real-world samples can be formulated as described in Eq (3).


$$\begin{array}{c} Z=\mu +\varepsilon *\sigma \end{array}$$ (3)

In this formulation, μ denotes the mean vector, ε represents a random sample from a standard normal distribution, and σ corresponds to the standard deviation vector.

**Fig 3:**
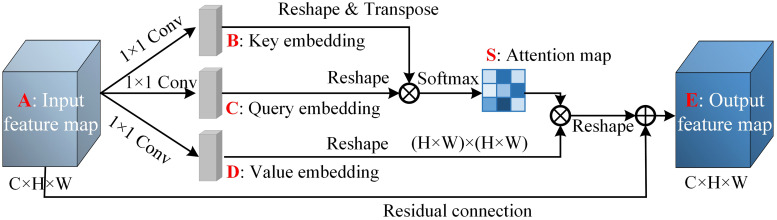
Schematic diagram of the structure of the PA mechanism module [29].

Since the standard VAE model has limited ability for conditional data generation, this study employs an enhanced variant, the CVAE [28]. The CVAE framework offers significant advantages by enabling data generation conditioned on specific input variables, thereby establishing a methodological basis for addressing the challenges of dynamic welding current data updating in this research. The CVAE extends the VAE architecture by incorporating conditional variables into both its encoder and decoder networks, ensuring that the generated data conforms to predefined conditions. Once trained, the CVAE decoder can generate corresponding welding current profiles based on specified input labels, such as welding parameters and electrode states. However, its performance in generating high-dimensional and complex temporal data can be suboptimal, often resulting in output signals that lack sharpness or exhibit bias. To overcome this limitation, a PA module [29] was further introduced. The core functionality of the PA mechanism lies in its capacity to capture long-range spatial dependencies across feature maps, irrespective of relative positional distances, meaning that similar features maintain correlations throughout the entire spatial domain. By integrating this PA mechanism into the CVAE framework, the enhanced model demonstrates improved sensitivity to discriminative features across different current waveforms. The structure of PA is shown in Fig 3.

**Fig 4:**
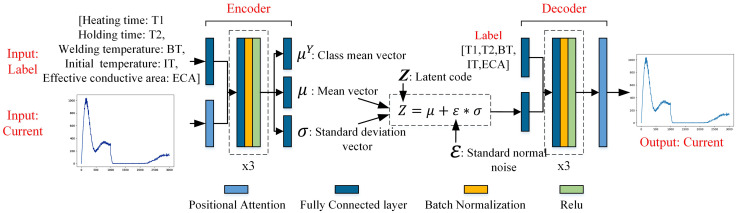
Schematic diagram of the PA-CVAE principle and model structure.

As illustrated in Fig 3, an input feature map A, with dimensions C × H × W, is first passed through three parallel convolutional layers to generate feature maps B, C, and D. These feature maps are then reshaped into two dimensional matrices of size C × N, where N = H × W denotes the total number of spatial positions. To compute the attention weights, the reshaped feature map B is transposed and multiplied with the reshaped feature map C, followed by a softmax activation function to yield the normalized weight matrix S. Subsequently, the reshaped feature map D is matrix-multiplied by the transposed weight matrix S, enabling each spatial position in feature map D to aggregate features from other positions based on the learned correlation information in S, thereby incorporating richer contextual information. A coefficient α then scales the resulting matrix, reshaped back to the original dimensions C × H × W, and combined with the original input feature map A via residual addition to produce the final output feature map E. By integrating the PA module at both the encoder and decoder stages of the CVAE, specifically after the encoder output and before the decoder output, the model gains enhanced sensitivity to discriminative features across different current waveforms. This significantly improves the representation capability of the CVAE framework, resulting in more accurate and robust current data generation. The detailed network architecture is presented in Fig 4.

The CVAE presumes that samples belonging to the same class share an identical exclusive mean μ^Y^, and aspires to regulate the model output by manipulating the class mean μ^Y^. This objective is accomplished by incorporating label data Y into the input layers of the encoder and decoder and modifying the Kullback-Leibler (KL) loss. The modified KL loss expression is presented in Eq (4).


$$\begin{array}{c} L_{\mu ,\sigma^{\text{2}}}=\frac{\text{1}}{\text{2}}\sum_{i=\text{1}}^{d}\left[{\left(\mu_{i}-\mu_{i}^{Y}\right)}^{\text{2}}+\sigma_{i}^{\text{2}}-log\sigma_{i}^{\text{2}}-\text{1}\right] \end{array}$$ (4)

Fig 4 shows that the encoder of the CVAE takes labels and current curves as inputs. The labels encompass the heating time (T1), holding time (T2), welding temperature (BT), initial electrode temperature (IT), and effective conductive area (ECA). First, the welding parameters traverse the first fully connected layer (FC), which consists of 1024 neurons, to increase the elevation of the feature dimension. Subsequently, it is concatenated with the current curve data weighted by the PA module and fed into the three-layer neural network module. Each layer within this neural network module comprises: “FC + batch normalization (BN) + ReLU activation function,” with 1024 neurons in the FC layer. Then, through three independent FC layers (each consisting of 20 neurons), the predictions of the category mean μ^Y^, the sample mean μ, and the logarithm of sample variance logσ^2^ are obtained. Here, logσ^2^ is selected to be predicted directly instead of variance σ^2^ because σ^2^ is invariably nonnegative and demands additional activation function processing, whereas logσ^2^ can be either positive or negative.

According to the dimensions of μ and logσ^2^, ε of the same dimension is randomly sampled from the standard normal distribution, and then the decoder input Z is obtained by applying Eq (3). After Z undergoes a layer of the FC layer to achieve feature dimension elevation and is concatenated with the welding parameter features that have also undergone dimension elevation, it is then input into the same three-layer neural network module as in the encoder, and finally corrected by the PA module to obtain the predicted current curve.

In the self-updating simulation step of the electrode crack model, when a crack expands and the electrode current curve under the current ECA needs to be generated since Z follows the standard normal distribution, it is necessary to randomly sample from the standard normal distribution, integrate it with the welding parameters and the ECA, and input it into the trained decoder to generate the current curve corresponding to the current state. This current is then applied to both ends of the TCB electrode geometric model to accomplish the current update in the crack expansion simulation process.

### Method for constructing a 3D crack prediction model for the electrode

**Fig 5:**
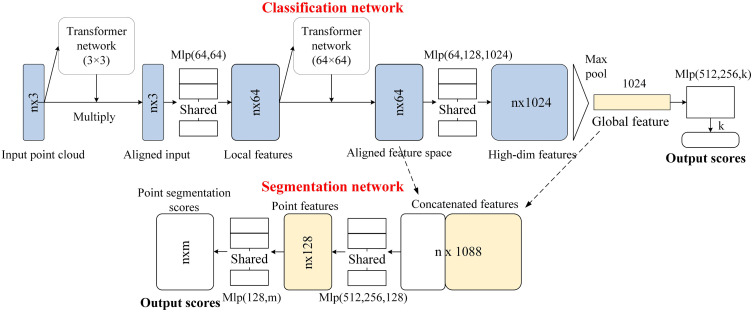
Model structure of PointNet [31].

Since the electrode simulation generates PHILSM point cloud data, PointNet [31], a deep learning model specifically designed for processing such data, was selected for predicting electrode cracks. Traditional convolutional neural network (CNN) excels at handling regular pixel grid data but is less effective for unordered and irregular point cloud data. The primary innovation of PointNet is its ability to extract representations directly from point cloud data without needing to convert it into regular 3D voxel grids or other structured formats. Its model architecture is shown in Fig 5.

The PointNet architecture comprises two core components: a feature transformation network and a classification/segmentation network. The model takes an n × 3-dimensional matrix as input, representing 3D coordinates of n points, and employs two transformer networks to learn 3 × 3 and 64 × 64 affine transformation matrices, respectively. This design ensures rotation and translation invariance while optimizing the feature space. A shared multilayer perceptron (MLP) progressively elevates local features to 1024-dimensional representations, with global feature vectors extracted via max pooling for classification tasks. For segmentation tasks, these global features are concatenated with each point’s low-level features and further processed through MLPs to generate per-point segmentation results. Notably, the model exhibits permutation invariance to input point ordering and can process point clouds of arbitrary sizes, demonstrating both computational efficiency and robustness in 3D data processing.

**Fig 6:**
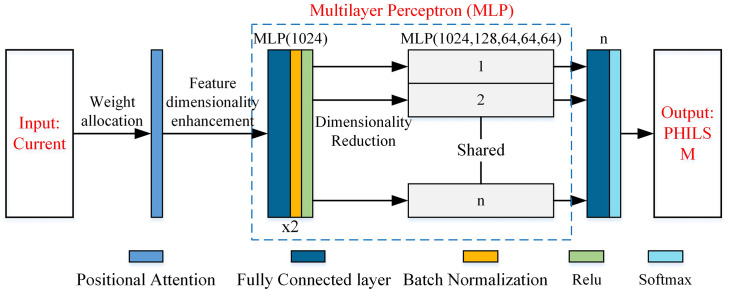
Model structure of PA-RePointNet.

Given that the model input data for this study consisted of current profiles, a PA mechanism was incorporated into a modified PointNet-like architecture to assign weights to current values at different temporal positions. In the PA-RePointNet model proposed herein, “Re” signifies reversal or inversion, reflecting its conceptual opposition to the PointNet model’s implementation approach. Specifically, PA-RePointNet utilized current profile data as its input. Subsequently, after a series of feature dimensionality augmentation and reduction operations, it predicted the PHILSM values (forming a PHILSM point cloud) at each finite element node associated with the crack. This process thereby enabled the prediction of 3D thermomechanical fatigue cracks in the electrode. The transformer network within the original PointNet architecture is designed to achieve geometric transformation invariance for point cloud data. However, because PA-RePointNet predicted PHILSM values for each crack-related finite element node in a fixed sequence, the transformer network was not incorporated. The final architecture of the designed PA-RePointNet model is illustrated in Fig 6.

Specifically, as depicted in Fig 6, the current profiles were initially input into the PA module. Following weight assignment, the data were then fed into the first MLP module. Each layer within this MLP module consisted of an “FC layer + BN + ReLU activation function,” where the number of neurons in the FC layer was 1024, facilitating feature dimensionality enhancement. This configuration is analogous to the K-classification MLP module in the PointNet model. In the PointNet classification model, before point cloud data enters the classification MLP module, each feature of the point cloud data is first elevated to 1024 dimensions via a shared MLP module. Subsequently, a max-pooling operation is applied to these 1024-dimensional features to achieve effective feature extraction and dimensionality reduction. Given that PA-RePointNet is conceptualized as an inverse model of PointNet, it incorporates a series of operations after the initial MLP. Specifically, after the data passed through the first MLP, feature dimensionality enhancement mapping and subsequent dimensionality reduction operations were performed for each feature vector corresponding to a point. The first layer of the second MLP module elevated the features of each point to 1024 dimensions, after which they were progressively reduced to 64 dimensions. The same replica of the MLP was utilized for all input data streams to ensure consistent processing. Following processing by the shared MLP module, an FC layer was applied, followed by a softmax activation function, which constrained the model’s output values to the range of [−1, 1]. Ultimately, this process yielded the PHILSM values for each finite element node. This methodology effectively enhanced the accuracy of both feature extraction and output generation.

## Results and analysis

### Simulation analysis of electrode crack propagation behavior based on the DT model

#### Evaluation of temperature simulation results.

This section presents a comparative analysis between experimentally measured temperature field data from crack-free TCB electrodes and corresponding simulation results to validate the high fidelity of the developed thermal simulation model. The experimental temperature field evolution during the entire welding process was captured using an infrared thermal imager, while the physical current data obtained from experiments served as the energy input for the Abaqus simulation model.

**Fig 7:**
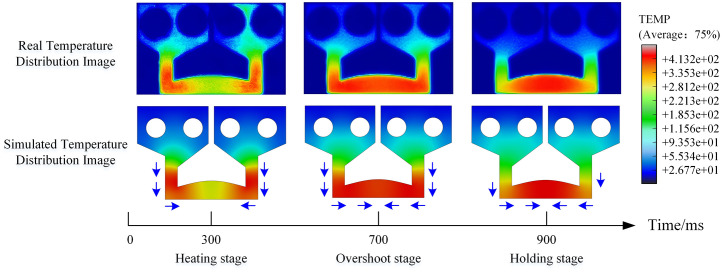
Real and simulated temperature distributions of TCB at welding stages.

Fig 7 compares the simulated and actual thermal images of the electrode during different welding stages. Both simulation and experimental results demonstrate highly consistent temperature evolution patterns during the transition from heating to holding stages, showing remarkable similarity in thermal distribution and heat transfer behavior. During heating, distinct heat accumulation is observed at both ends of the electrode in both simulated and actual images. As welding progresses, the high-temperature zone gradually migrates toward the central region. These results conclusively validate the high fidelity of the developed numerical model, establishing a solid foundation for subsequent DT-based crack prediction.

**Fig 8:**
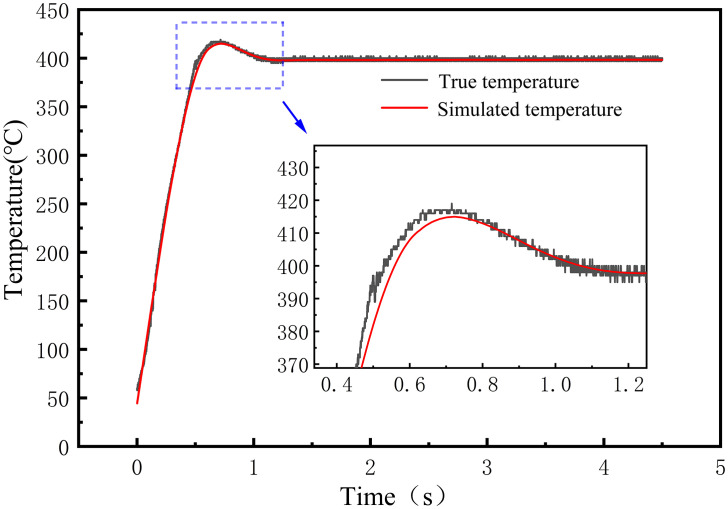
Simulated and real temperature curves at the center position of the TCB electrode.

To further validate the fidelity of the temperature simulation, a pointwise comparison was performed between simulated and measured temperature histories at the electrode thermocouple location, as shown in Fig 8. The two curves showed similar overall trends. During the initial heating stage, the simulated temperature was slightly lower than the measured temperature. As the process entered the later heating stage, the agreement improved markedly. In the overshoot stage, the simulated response lagged the measurement slightly. Once the dwell stage began, the two curves showed good agreement. These results indicate that the model reproduces the dynamic evolution of temperature during welding with satisfactory accuracy.

Root mean square error, RMSE, is a widely used statistic for quantifying the deviation between predicted and observed values. By squaring individual errors, it assigns greater weight to larger deviations and reflects both the accuracy and the stability of predictions [32]. Because RMSE is sensitive to outliers, it is suitable for evaluating numerical simulations that exhibit transient fluctuations. Accordingly, to quantify the accuracy of the thermal simulation model, the RMSE between the simulated and measured temperatures was computed, and the results are reported in Table 2.

**Table 2:** RMSE of simulated and measured temperatures at different welding stages.

Stage	Time (ms)	RMSE (°C)
Heating stage	0-500	10.0
Overshoot stage	501-1000	5.9
Holding stage	1001-4500	4.1
Overall	0-4500	5.3

The results show that the RMSE during heating was 10.0 °C, slightly higher than in the other two stages. The difference mainly reflects the rapid temperature rise during heating and the pronounced transient variations in heat input and conduction, which introduce a small lag between the simulated and measured responses. Overall, the model achieved an RMSE of 5.3 °C. Relative to a welding temperature of 420 °C, this corresponds to approximately 1.26%. These findings indicate that the thermal simulation model attains high predictive accuracy and stable performance across the welding stages and captures the dynamic evolution of the temperature field during welding.

#### Analysis of the crack initiation location and propagation direction.

The XFEM-based crack propagation simulation requires careful selection of an appropriate damage criterion, specifically either the maximum principal stress criterion or the maximum principal strain criterion. Additionally, for specific simulation objects, initial crack locations may need to be predefined to ensure that crack propagation originates from expected regions. As evidenced in Fig 7, significant temperature variations occur in the lower-middle and side regions of the electrode during welding. The resulting thermal expansion induces stress concentrations at transitional zones of the electrode structure. Theoretically, simulations employing the maximum principal stress criterion would predict crack initiation at these structural transition zones (where maximum principal stress concentrates), followed by propagation along the descending stress gradient. However, actual welding observations revealed discrepancies between real fracture locations in this electrode type and predictions based on the maximum principal stress criterion, demonstrating its inadequacy as a damage criterion for TCB electrode crack simulations.

Moreover, in actual welding processes, brittle fractures of TCB electrodes predominantly occur in the negative electrode region. This phenomenon stems from the unidirectional current flow characteristic of monopolar power supply systems, where electron migration leads to polarity-specific charge accumulation. Such electron accumulation intensifies inter-electron collisions, causing thermal stresses to initially develop and concentrate at the negative pole. This localized stress concentration disrupts thermal equilibrium, ultimately triggering fracture failure at the negative electrode. During fracture initiation, the separation between fracture surfaces often generates arc discharges through air breakdown, producing localized, ultra-high temperatures that melt the electrode material. Consequently, the final fracture surface typically exhibits a smooth, molten metallic droplet morphology. To accurately predict and understand the fracture behavior of TCB electrodes, a comprehensive analysis of both thermal strain evolution and maximum principal strain distribution during welding is essential. This approach enables the reliable determination of crack initiation sites and propagation paths under operational conditions.

**Fig 9:**
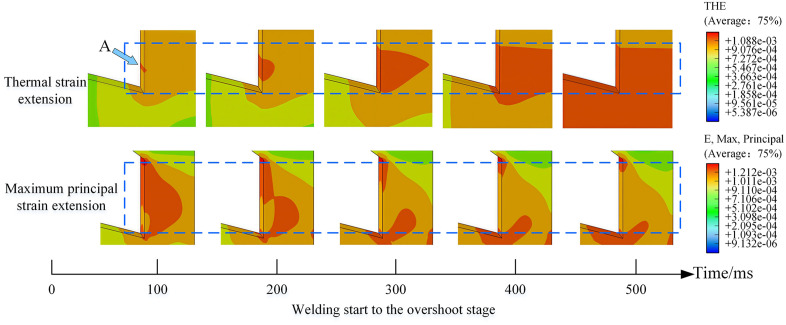
Schematic illustration of TCB thermal strain and maximum principal strain evolution.

The first row of Fig 9 demonstrates the thermal strain diffusion behavior in the negative electrode during the heating process. As shown in the figure, thermal strain initially nucleates at the lower inner region of the negative electrode (indicated by Point A) and gradually propagates outward, eventually penetrating the entire side of the electrode before further extending toward the central region. Throughout the welding process, the heating phase is characterized by its short duration and rapid temperature variation. Since the temperature on both sides of the electrode decreases quickly after entering the overshoot stage, the region around Point A exhibits particularly rapid temperature changes and significant thermal strain. This observation aligns with the expected thermomechanical response of the electrode material under such transient thermal loading.

Thermal fatigue damage typically initiates not only at stress concentration zones but also in regions with maximum thermal strain on the workpiece surface [33]. When the surface thermal strain of the workpiece exceeds the material’s elastic limit at elevated temperatures, thereby entering the plastic strain regime, minute localized plastic deformation occurs. Furthermore, during thermal cycling, the material repeatedly traverses its ductile-to-brittle transition temperature. Concurrently, under cyclic mechanical stress, intergranular fissures may also develop during the material’s expansion and contraction phases. The accumulation of such micro-damage ultimately culminates in the formation of fatigue cracks oriented perpendicularly to the workpiece surface. Once formed, these fatigue cracks propagate inward under the combined influence of cyclic thermal and mechanical stresses, eventually leading to fracture. Moreover, intensified oxidation processes within these fissures can accelerate crack propagation. Therefore, it was posited that micro-damage would first initiate at point A, subsequently developing into a crack, which would ultimately cause the fracture failure of the TCB electrode under cyclic thermal and mechanical stresses.

The second row of Fig 9 presents the evolution of maximum principal strain distribution in the TCB electrode during welding. The results reveal that during the heating phase (0–500 ms), a distinct strain distribution, oriented toward the upper right, gradually develops in the lower inner region of the negative electrode and subsequently diminishes. This observation indicates that the combined effects of electrode geometry and thermal strain induce a deformation tendency directed toward the upper right in this region during heating. The deformation pattern likely results from constrained thermal expansion, where the heated central portion of the electrode attempts to expand laterally but is restricted by the flanking structures, thereby creating a propensity for crack initiation.

In summary, through a comprehensive analysis of thermal strain and maximum principal strain evolution in TCB electrodes during welding, combined with thermal fatigue damage mechanisms and the electrothermal characteristics of the negative electrode region, it is confirmed that initial cracks nucleate at Point A in Fig 9 and propagate along the direction of maximum principal strain toward the upper right.

#### Analysis of 3D crack extension results after TCB electrode updating.

**Fig 10:**
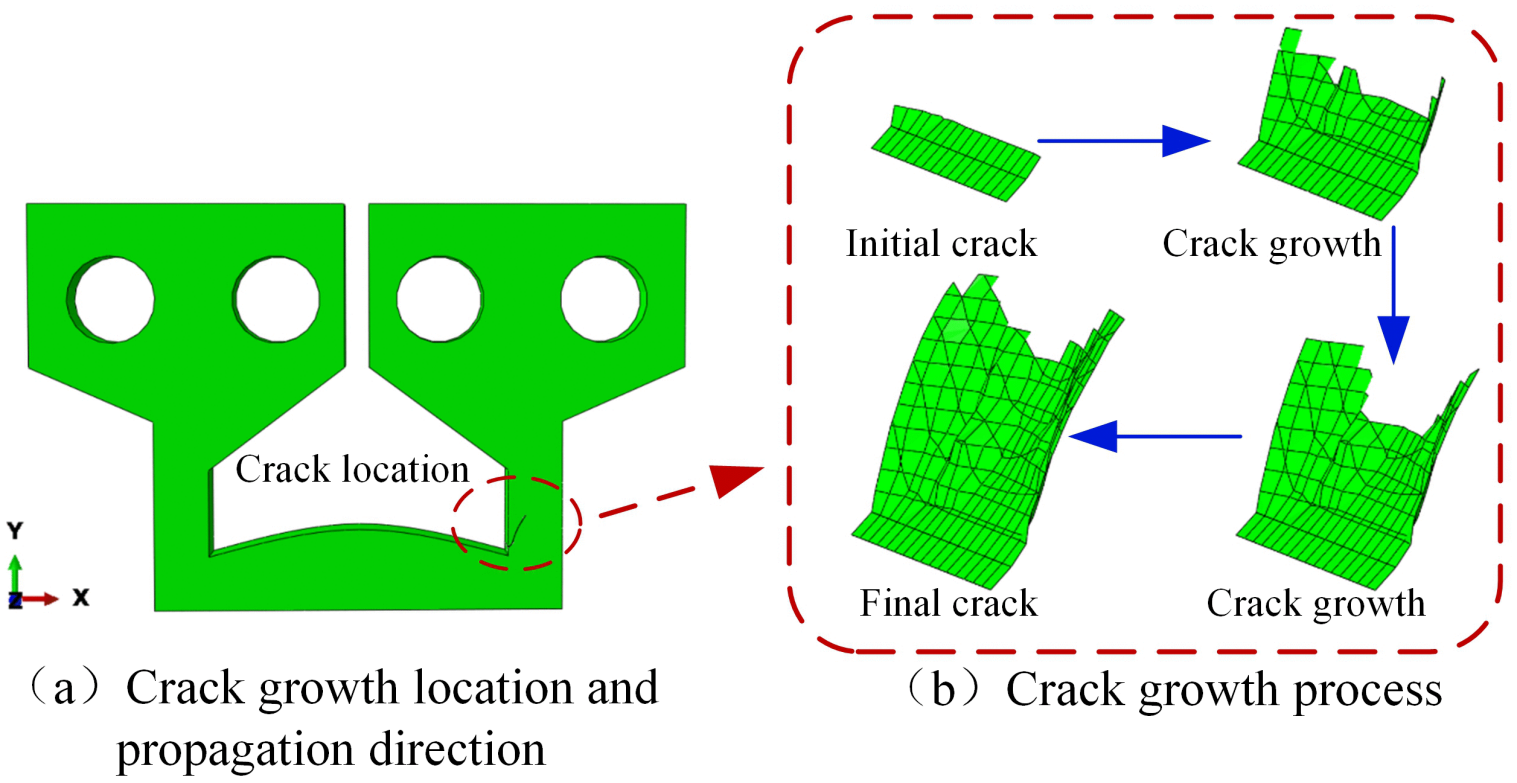
Simulation results of 3D thermomechanical fatigue crack propagation in the TCB electrode.

Based on the stress simulation model, an initial planar crack component, 0.1 mm in length and 1.6 mm in width, was predefined. This component was then rotated to form a 45° angle with the horizontal direction and subsequently assembled with the TCB electrode’s geometric model at location A, as depicted in Fig 9. Following this, by employing the model self-updating method, the 3D thermomechanical fatigue crack propagation simulation results for the TCB electrode were ultimately obtained, as presented in Fig 10.

Firstly, Fig 10(a) clearly illustrates the final crack propagation direction. This result was highly consistent with the analytical conclusions based on the maximum principal strain criterion, thereby indirectly corroborating the accuracy and reliability of the simulation process. Fig 10(b) depicts the crack growth process at the initiation site. During crack growth from initiation to fracture, the complex and irregular crack morphology means that conventional metrics such as crack length or area are inadequate for effectively quantifying its propagation state. Consequently, the ECA was introduced to characterize different crack states. As shown in Fig 10, given that the 3D coordinates of the nodes of the crack tip elements in each layer perpendicular to the XY plane, as well as the electrode’s geometric parameters, were known, the perpendicular distance from the crack tip elements of each layer to the right side surface of the electrode could be determined. Furthermore, as the length of each element was known to be 0.1 mm, the ECA was calculated by summing the areas of the vertical rectangular cross-sections formed by each layer of crack tip elements (perpendicular to the XY plane) extending in the X-direction to the right side surface of the electrode. Through this calculation, 44 distinct ECA values were obtained.

**Fig 11:**
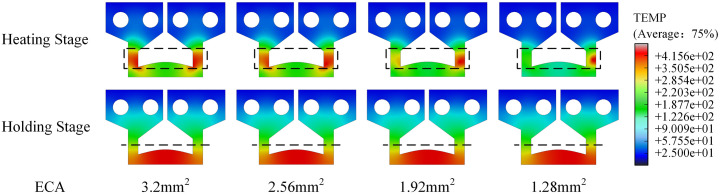
Simulation results of TCB temperature distribution with different ECAs in the heating and holding stages.

Under different ECA values, the temperature field exhibited discernible variations. Electrode temperature distribution images for different crack morphologies are presented in Fig 11. As the crack propagated, corresponding to a decrease in ECA, the initial heating location on the electrode shifted towards the upper right, following the crack tip. This phenomenon was particularly conspicuous during the heating-up stage. Consequent to this shift, the temperature distribution on the welding face during the holding stage no longer exhibited central symmetry relative to the electrode’s structure. Instead, it displayed a trend of being lower on the left and higher on the right (crack tip side). In the actual welding process, as a crack extends, the presence of fissures and oxides within the crack leads to a reduction in the cross-sectional ECA. This, in turn, causes the initial heating location on the electrode to gradually shift towards the upper right, tracking the crack tip. This observation aligns with the simulation results, further substantiating the accuracy of the constructed simulation model.

**Fig 12:**
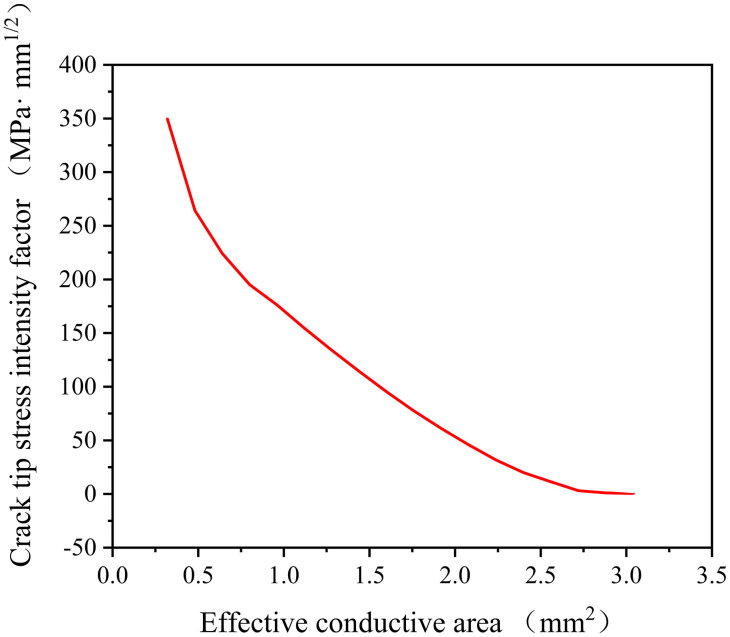
Crack tip SIFs for different ECAs.

The SIF at the crack tip serves as both a mechanical parameter for crack propagation and a fracture criterion. Its accurate calculation under different crack morphologies is crucial for simulating thermal fatigue crack growth in TCB electrodes. Fig 12 presents the SIF results for varying ECA values. As shown in Fig 12, the crack tip SIF exhibits a negative correlation with ECA, meaning fracture risk increases with crack extension. Specifically, when the electrode’s ECA decreases to 0.48 mm^2^, the SIF reaches 264.42 MPa·mm^1/2^, exceeding the material’s fracture toughness of 252.98 MPa·mm^1/2^. This indicates fracture initiation, satisfying the simulation termination criterion.

### Current data augmentation for electrode fatigue damage based on PA-CVAE

#### Current data set construction.

Based on custom-made TCB electrodes and the previously determined crack initiation site, initial defects oriented at 58° towards the upper right, with lengths ranging from 0.0 to 3.0 mm (at 0.1 mm intervals), were predefined. Each defect length corresponded to a specific ECA, and a total of 31 such electrodes were fabricated. Through testing conducted at an ambient temperature of 10 °C, it was determined that if the interval between two welding operations was 10 s, the initial electrode temperature stabilized at approximately 115 °C; if the interval was 15 s, it stabilized at approximately 105 °C. Given that the interval between welding operations in actual production processes is typically neither excessively long nor short, an initial electrode temperature range of 80–130 °C was selected for testing. With a 5 °C interval, 11 data points were thus obtained for each defect length. Consequently, a total of 341 data points were acquired from these 31 custom-made electrodes. Of these, 80% were randomly allocated to the training set, and the remaining 20% were assigned to the test set.

#### PA-CVAE model training.

**Fig 13:**
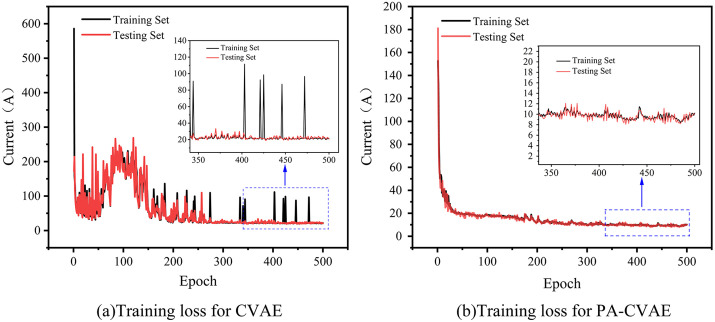
Training loss for the CVAE vs PA-CVAE.

The CVAE and PA-CVAE models were trained using the current data set, with both models employing Mean Absolute Error (MAE) as the loss function to enhance training performance. The MAE is a commonly used statistical metric that measures the average absolute difference between the predicted and observed values. A lower MAE indicates that the expected results are, on average, closer to the actual observations [34]. In the PA-CVAE model, the Adam optimizer was selected to replace the RMSProp optimizer used in the CVAE model, aiming to stabilize the training process. The learning rate was set to 0.001. Fig 13 presents the MAE loss curves for the current prediction during the training process of both models.

**Fig 14:**
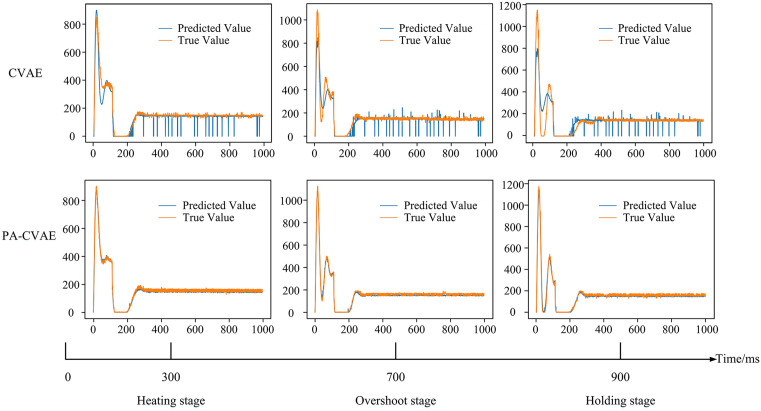
Comparison of CVAE and PA-CVAE curve fitting on test data.

Fig 14 presents the current curve fitting results between the CVAE and PA-CVAE models on the test set after training completion.

First, as shown in Fig 13(a), the unmodified CVAE converges at approximately 270 epochs with a relatively fast rate, yet two clear deficiencies remain. The loss curve exhibits pronounced fluctuations during training, which indicates instability, and the final convergence value is comparatively high. Second, the first row of Fig 14 further confirms the limitations of the CVAE. Across test cases with different peak currents, the CVAE yields unsatisfactory current predictions in the heating stage, with substantial deviations from the measured values. As the current rises rapidly, its predictive capability in the overshoot stage becomes severely inadequate, and the predicted current traces fit the measured curves very poorly. Although performance improves in the dwell stage, the predictions display a “vertical streak” artifact, that is, the model outputs discontinuous discrete values at certain time steps. These results indicate that the unmodified CVAE cannot be directly applied to the current dataset.

For the PA-CVAE, Fig 13(b) shows convergence at approximately 400 epochs with a similarly fast rate. Compared with the CVAE, PA-CVAE exhibits a more stable training trajectory and a lower terminal loss on average. This improvement is attributed to the use of the Adam optimizer together with MAE as an auxiliary loss for current prediction. In addition, the second row of Fig 14 indicates that PA-CVAE achieves good fits for current traces with different peak values across all stages, and it effectively eliminates the “vertical streak” artifact observed in the dwell stage of the CVAE results. This enhancement primarily arises from the PA module, which enables the model to capture temporal positional features of distinct current profiles more sensitively. To further quantify performance, the MAE values of the two models and their relative improvements were computed and summarized, as reported in Table 3.

**Table 3:** Comparison of the prediction performance of CVAE and PA-CVAE models.

Model	MAE (A)	Relative improvement
CVAE	21	–
PA-CVAE	9	59.2%

From the table, the MAE of the PA-CVAE model was 9 A, whereas the MAE of the CVAE model was 21 A, corresponding to an improvement in predictive accuracy of about 59.2%. Because the measured peak welding currents across different electrode defect lengths ranged from 692 A to 1242 A, the resulting relative prediction error was approximately 0.7% to 1.3%. These results indicate that the model can stably generate high-fidelity welding current data under varying electrode defect and initial temperature conditions, and they further validate the effectiveness of the PA module on current-based datasets.

### 3D crack prediction of electrodes based on the PA-RePointNet model

#### Crack data set construction.

The PHILSM point clouds under varying ECA values were obtained by dynamically replacing the initial current in the model self-updating process with current data generated by the PA-CVAE model. A data set was constructed by matching the generated current data with PHILSM point clouds sharing identical ECA values. The simulation yielded 44 distinct PHILSM point clouds in total, with each point cloud representing a unique ECA. Each PHILSM point cloud was paired with current data corresponding to 11 different initial electrode temperatures, resulting in a comprehensive data set containing 484 data points. The data set was randomly divided, with 80% allocated to the training set and the remaining 20% assigned to the test set.

#### PA-RePointNet model training.

The training objective of the PA-RePointNet model is to minimize the prediction error of PHILSM values within the test set. The model employs MAE as the loss function for PHILSM prediction and was trained using the Adam optimizer with a learning rate set to 0.001. It is noteworthy that the simulation model employed in this study is characterized by high precision. Given that the PHILSM value of each finite element node represents the distance from that node to the crack surface, nodes in extreme proximity to the crack surface may exhibit PHILSM values smaller than 1e-5. Such exceedingly small PHILSM values make precise prediction difficult, posing a challenge to model training. Furthermore, while reducing the learning rate to enhance prediction accuracy is a feasible approach, it significantly decelerates the training process. To effectively address this issue, the PHILSM values within the constructed dataset were uniformly scaled up by a factor of 1000.

**Fig 15:**
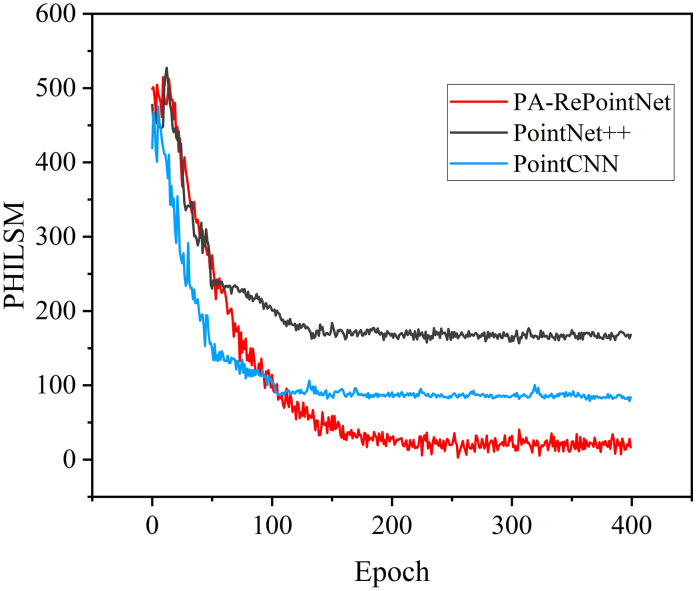
MAE loss curves of PA-RePointNet, PointNet++, and PointCNN.

To evaluate the performance of PA-RePointNet, two representative point-cloud deep learning models, PointNet++ [35] and PointCNN [36], were used as comparators. These models are widely applied to feature extraction and geometric learning for structural damage identification and crack prediction. The three models were trained and tested on the same dataset with the same loss function and hyperparameters. Fig 15 shows the MAE loss curves.

As shown in Fig 15, the MAE for all three models fell rapidly at the start of training and then approached a steady value as iterations increased. PointNet++ converged quickly in the early stage but leveled off at a higher loss. PointCNN declined at a moderate rate and stabilized after about 100 iterations. In contrast, PA-RePointNet decreased more slowly at first and reached its minimum near iteration 260, with a loss clearly lower than those of the other two models. This pattern indicates that PA-RePointNet, although slightly slower to converge at the beginning than PointNet++, exhibited stronger sustained learning and better overall convergence. Errors continued to decline in the later stage of training, which suggests superior capability in feature extraction and spatial relationship modeling. The improvement mainly arose from the PA module, which strengthened spatial dependency learning across point clouds and enabled more accurate localization of the crack tip and its surrounding geometry. As a result, overall prediction accuracy and stability improved.

To further compare the predictive performance of the three models, goodness of fit and prediction accuracy were evaluated using the coefficient of determination (R²) and RMSE. R² quantifies the agreement between predictions and ground truth, and larger values indicate a better fit [37]. The results on the test set are summarized in the table below.

As presented in Table 4, PA-RePointNet achieved an MAE of 2.8 and an RMSE of 5.1, both lower than those of PointCNN and PointNet++. These results indicate smaller prediction errors and greater robustness. The R² value reached 0.9378, reflecting excellent goodness of fit and a strong capability in capturing spatial features. In comparison, PointCNN achieved an R² of 0.8264. It tracked the overall trend but showed limitations in regions with complex crack geometry. PointNet++ yielded an R² of 0.5476, indicating weaker modeling of crack spatial relationships and a poorer fit. Overall, PA-RePointNet outperformed the baseline models in accuracy, goodness of fit, and stability, confirming the effectiveness of the proposed approach for crack point-cloud prediction.

**Table 4:** Comparison of the prediction performance of PA-RePointNet, PointCNN and PointNet++ models.

Model	MAE	R^2^	RMSE
PA-RePointNet	2.8	0.9378	5.1
PointCNN	8.1	0.8264	12.3
PointNet++	17.1	0.5476	19.9

**Fig 16:**
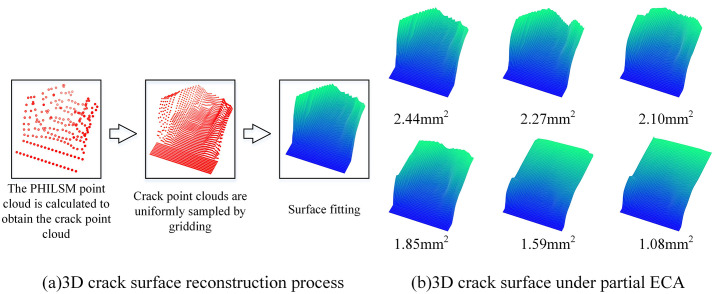
Process of fitting a PHILSM-based point cloud onto a 3D crack surface.

The PHILSM point cloud data predicted by PA-RePointNet consists of discrete finite element nodes. Each node solely carries information about its distance to the crack surface (the PHILSM value) and cannot directly describe the continuous crack surface morphology. Therefore, calculation and interpolation are required to perform surface fitting on the PA-RePointNet-predicted PHILSM point cloud. This allows for the reconstruction of a smooth, continuous crack topology, subsequently yielding a 3D irregular crack surface. The reconstruction process is illustrated in Fig 16(a). During this procedure, the method in the model self-update was employed to compute a crack point cloud from the PHILSM point cloud. Interpolation was then applied to this crack point cloud to ensure that the final fitted crack surface was smoother. Fig 16(b) displays some of the 3D crack surfaces reconstructed after crack prediction.

To validate the proposed crack prediction method, crack prediction experiments were further conducted. Initial defect lengths of 0.9 mm, 1.1 mm, 1.3 mm, 1.6 mm, 1.9 mm, and 2.5 mm correspond to ECAs of 2.44 mm², 2.27 mm², 2.10 mm², 1.85 mm², 1.59 mm², and 1.08 mm², respectively. First, welding experiments were performed on electrodes with these initial defect lengths. Then, the relevant current data and initial temperatures were input into the trained PA-RePointNet model to predict the corresponding PHILSM point clouds and subsequently generate 3D crack surfaces for crack length measurement. To mitigate stochastic variation in model predictions, the model was executed independently ten times. Prediction accuracy and stability were assessed by computing the mean, standard deviation, and relative error across runs. The experimental results are shown in Table 5.

**Table 5:** Crack length prediction results with different preset defect lengths.

Preset defect length (mm)	Predicted average crack length (mm)	Standard deviation	Relative error (%)
0.9	0.8934	0.0134	0.74
1.1	1.0871	0.0354	1.18
1.3	1.3243	0.0167	1.87
1.6	1.6131	0.0278	0.82
1.9	1.9214	0.0217	1.13
2.5	2.5257	0.0361	1.03

As ECA may not intuitively reflect electrode fatigue damage in practical scenarios, crack length was employed here as a more intuitive metric for characterizing the crack state. Given that the preset defects on the electrodes were straight grooves fabricated by wire cutting, the predicted crack length for this validation was defined as the average distance from points on the predicted 3D front to the crack initiation site. As exhibited in Table 5, the mean predictions for all six cases were in close agreement with the true defect lengths, with a maximum relative error of 1.87%. Across the cases, the standard deviations ranged from 0.0134 to 0.0361 mm, indicating good stability and consistency during repeated measurements. These results further validate the high accuracy and robustness of the PA-RePointNet model for processing PHILSM point clouds.

To enable real-time performance evaluation, a monitoring application was developed using the PyQt5 framework. This software acquires real-time current, temperature, welding parameters, and system settings via an RS485 serial interface. After each welding process, the processed data are fed into PA-RePointNet to predict the corresponding PHILSM point cloud. The 3D crack surface is then reconstructed following the workflow illustrated in Fig 16. The system supports local inference with PA-RePointNet and provides functions for welding history management and customizable data storage. The final system was tested on the platform specified in Table 6.

**Table 6:** Test platform information for developed systems.

Item	Version
OS	Windows 11 Professional
CPU	AMD Ryzen 9 7945HX
GPU	NVIDIA GeForce RTX 4060 Laptop (8G)
Python	3.7.16
TensorFlow	2.7.0
CUDA	11.2
cuDNN	8.9.7

Using the developed monitoring system, the response time of PA-RePointNet in local mode was tested to assess compliance with strict production timing requirements. Timing started when the client received RS485 serial data and ended when the update of the 3D crack surface on the client interface was completed. This interval was defined as the system response time. Transmission delays of current and temperature signals from the welding system were excluded from the test analysis. Five rounds of tests were conducted, with 400 trials performed per round. The results are summarized in Table 7.

**Table 7:** The system response time of the PA-RePointNet model in local deployment modes.

Round	Maximum response time (ms)	Minimum response time (ms)
Round 1	810.98	507.28
Round 2	813.09	561.34
Round 3	839.81	563.63
Round 4	811.30	567.68
Round 5	778.74	559.83

As shown in Table 7, in the local-mode tests, the maximum system response time across five rounds was 839.81 ms, the minimum was 507.28 ms, and the average was 603.40 ms. Specifically, PA-RePointNet was required to predict the PHILSM values at each finite element node, and the crack surface was reconstructed according to the workflow illustrated in Fig 16. These two computations accounted for most of the time consumed per response. In production, the welding interval typically ranges from several seconds to tens of seconds. Within this window, a response time below one second met the real-time requirement for online monitoring of thermomechanical fatigue cracks in TCB welding electrodes.

In summary, the proposed DT-based prediction framework exhibits both high accuracy and computational efficiency. With an average computation time of 603.40 ms, it can satisfy the real-time demands of industrial welding processes.

## Discussion

This study proposed a DT-based method for 3D crack prediction in TCB electrodes and addressed the key challenge of online prediction of thermal fatigue cracks in electrodes. The central advance lay in combining a high-fidelity, automatically updated DT model, a data augmentation strategy based on PA-CVAE, and a 3D point cloud prediction model, PA-RePointNet. This integration improved prediction accuracy and robustness. The model captured the complex crack evolution under coupled thermal and mechanical loading, and the adaptively updated virtual model provided real-time and precise predictions of crack growth. In addition, to mitigate the scarcity of crack data, the PA-CVAE generated physically consistent current data, which enhanced the predictive capability of the DT framework. PA-RePointNet accurately predicted crack point clouds and reconstructed crack geometry, providing high-resolution 3D information for quantitative defect assessment and remaining life estimation, and it readily integrated with online monitoring for intelligent maintenance in TCB manufacturing.

Compared to other studies [14–17] based on 1D and 2D data, this research achieved a refined representation of multi-scale features and the complex, time-evolving morphology of 3D cracks. The proposed PA-RePointNet accurately predicted PHILSM point clouds that describe 3D crack geometry and showed rapid, stable convergence during training relative to PointNet++ and PointCNN. After scaling PHILSM by a factor of 1000, the model attained an MAE of 2.8, an RMSE of 5.1, and an R² of 0.9378, indicating high predictive accuracy. Applying interpolation and surface fitting to the predicted point clouds yielded smooth and continuous reconstructions of irregular 3D crack surfaces. These results verified the accuracy and reliability of the model for predicting 3D thermal-fatigue cracks in TCB electrodes and opened new opportunities for predictive maintenance and fault prevention in electronics manufacturing.

Despite substantial progress in electrode crack prediction, several limitations remain. First, the model assumes known and idealized material properties, which may deviate from the variations encountered in production. Aging, surface oxidation, and changes in the service environment can introduce uncertainty in electrode performance and reduce prediction accuracy. Second, the crack model focuses on thermomechanical fatigue. Its applicability to other modes, such as stress corrosion cracking (SCC) and brittle cracking, requires further validation. Finally, the computational cost is relatively high, and meeting stricter real-time requirements in high-frequency or large-scale production settings remains challenging.

Future research will proceed along several directions. First, in material modeling, a broader set of measured material parameters will be incorporated, and machine learning will be used to adapt constitutive models dynamically to variations in material behavior under different operating conditions. Second, the framework will be extended to additional crack modes, particularly SCC and brittle cracking, to increase generality. Third, computational efficiency will be addressed by improving prediction speed through parallel computing, model compression, and edge computing, so that stricter real-time requirements in high-frequency production can be met. In addition, integrating optimization methods with predictive modeling can improve computational efficiency and predictive accuracy and can enhance adaptability and robustness across diverse operating conditions [38].

## Conclusions

This study proposed a novel method based on DT technology for predicting 3D thermal fatigue cracks in TCB electrodes, addressing the complex challenges of online monitoring for electrode thermal fatigue cracks in the microelectronics packaging field. A multi-dimensional, multi-scale electrode DT entity was constructed. This virtual model, through an innovative adaptive updating strategy, can respond in real-time and with high accuracy to the material property degradation and damage accumulation of the physical electrode. Combined with the XFEM, it successfully simulated crack propagation behavior caused by complex electro-thermomechanical coupling during the welding process. The RMSE between the simulated temperature at the electrode thermocouple and the actual temperature was only 5.3 °C, with a percentage error of just 1.26%, robustly demonstrating the high precision of the electro-thermo model. The simulation results not only showcased the significant influence of crack morphology on temperature field distribution but also validated the model’s physical consistency by accurately identifying crack initiation sites and propagation directions.

To address the challenge of scarce high-value damage state data, this study proposes the PA-CVAE model, which integrates a PA mechanism to achieve precise generation of welding current data under varying ECA and initial temperature conditions. The results indicated that PA-CVAE far surpassed traditional CVAE in terms of prediction accuracy and stability, achieving a low convergence value of 9 A. This effectively addressed the current data updating problem and provided high-quality, diverse training samples for subsequent crack prediction models.

Building upon this foundation, the proposed PA-RePointNet model demonstrated the capability to output PHILSM values for each finite element node. After interpolation and surface fitting, these discrete point cloud data can accurately reconstruct complex 3D irregular crack surfaces. The PA-RePointNet model showed superior predictive performance relative to PointNet++ and PointCNN, achieving an MAE of 2.8, an RMSE of 5.1, and an R² of 0.9378. These values demonstrate high accuracy and robustness. Predicted 3D crack morphologies agreed closely with experimental results. The maximum standard deviation in crack length was 0.0361 mm, and the maximum relative error was 1.87%, confirming the reliability of the proposed approach. In addition, a monitoring system developed with PyQt5 verified that both local and cloud implementations of PA-RePointNet achieved response times below one second, indicating potential for real-time crack monitoring in industrial applications. Overall, this work provides a practical and accurate approach for real-time crack diagnosis and health management of TCB electrodes and contributes to predictive maintenance and intelligent manufacturing in electronic packaging.

This work not only provides a solid foundation for online health monitoring and crack suppression of TCB electrodes in the microelectronics packaging field but also, through the DT framework, opens up new avenues for predictive maintenance and intelligent manufacturing of critical components. Future research should focus on enhancing the computational efficiency and real-time performance of the models to meet the more stringent demands of high-frequency or large-scale production scenarios. The generalizability of the models will also be explored, aiming to apply them to a broader range of industrial fields, thereby delivering greater value in the health monitoring and intelligent manufacturing of more critical components.
